# The Effects of Hydration on Growth of the House Cricket, *Acheta domesticus*

**DOI:** 10.1673/031.008.3201

**Published:** 2008-04-21

**Authors:** Kevin E McCluney, Rishabh C Date

**Affiliations:** ^1^ School of Life Sciences, Arizona State University, Tempe, AZ 85287-4601; ^2^ University of California, San Diego, 9500 Gilman Dr., La Jolla, CA 92093

**Keywords:** individual growth, water stress, desiccation, size, dry mass

## Abstract

Maintenance of biochemical gradients, membrane fluidity, and sustained periods of activity are key physiological and behavioral functions of water for animals living in desiccating environments. Water stress may reduce the organism's ability to maintain these functions and as such, may reduce an organism's growth. However, few studies have examined this potential effect. The effects of altered hydration state of the house cricket, *Acheta domesticus* L. (Orthoptera: Gryllidae) on individual growth were studied under laboratory conditions. Crickets were permitted access to water for three different durations each day, resulting in significant differences in hydration state (32% greater hydration for maximum than minimum duration of water availability). Growth was 59% and 72% greater in dry mass and length, respectively, between the lowest and highest hydration state treatments. These findings may be representative for a variety of animal species and environments and could have important ecological implications.

## Introduction

From a biological perspective, individual growth is defined as an increase in body size or biomass over time. This biomass increase requires inputs of various chemical constituents of physical and structural biomolecules (Sterner and Elser 2002). For invertebrates, the elements of C, N, and P are most important, generally in the form of carbohydrates, lipids, and proteins (Chapman 1998; Sterner and Elser 2002). Additionally, water must be consumed during growth and for daily maintenance or else dehydration and water stress will result, as metabolic water production is usually insufficient to balance losses (Hadley 1994).

Dehydration and water stress are likely to depress biomolecular growth by reducing physiological performance in a number of ways. As a universal solvent and transport medium, water is essential to maintaining proper biochemical gradients and physiological homeostasis (Minnich 1982; Willmer et al. 2000; Alpert 2006). Dehydration can cause reduced efficiency of the electron transport chain and biochemical reactions (e.g. through hyperkalemia, Minnich 1982), as well as the production of destructive reactive oxygen species, the denaturing or clumping of proteins, the destruction of cellular membranes, and death (Chapman 1998; Alpert 2006).

In order to reduce the damage caused by dehydration, organisms allow the dehydration of certain pools of body water in favor of maintenance of others, or add increased effort to the regulation of electrolytes (e.g. K), a potentially costly compensatory mechanism (Minnich 1982). In addition, organisms produce a variety of sugars (e.g. trehalose), antioxidants, and proteins (e.g. heat shock proteins) that help modulate membrane fluidity and reduce oxidation (Willmer et al. 2000; Alpert 2006). Production of these molecules is also chemically and energetically costly and may divert resources away from growth (Alpert 2006). Furthermore, any damage that is caused due to dehydration must be repaired, adding additional chemical and energetic costs, potentially reducing growth. Finally, growth may be reduced by a need to conserve water, which causes lower activity rates and thus lower acquisition of other resources (Stamps 1976; Stamps and Tanaka 1981; Lorenzon et al. 1999). Water stress may also reduce growth when animals seek thermal environments at a lower temperature than that most conducive to biochemical processes as suggested by Lorenzon et al. (1999).

Studies of the effects of water availability and water stress on animals and plants have sometimes yielded variable results, although strong positive effects of water on growth of lizards have been well documented (Stamps and Tanaka 1981; Lorenzon et al. 1999) along with rodents (Kam and Degen 1994), turtle eggs (McKnight and Gutzke 1993), and insects (Floater 1997). Slansky and Wheeler (1991) found a negative effect of increased food hydration on growth of velvetbean caterpillars (*Anticarsia gemmatalis*). However, caterpillars were kept at ∼ 70% humidity and all diets contained a large amount of water (68% or 81%). Thus it is unlikely that these differences in food hydration had large impacts on the hydration of the caterpillars. They do note, however, that there were significant positive differences in caterpillar hydration only at median nutrient levels, but they do not provide magnitudes for differences. Without causing sufficient variation in hydration, it is likely that Slansky and Wheeler (1991) only witnessed the effects of variation in nutrient availability when water was not limiting. When nutrients are limiting, a negative relationship with food hydration would be expected, since more hydrated food has a lower nutrient content by dilution. Slansky and Wheeler (1991) did not attempt to examine the influence of water stress on growth, but their study serves as a good example of some of the factors that may contribute to the variability in responses observed in the literature. This variability may be attributable to four factors relating to water balance: variation in thresholds for water limitation/stress, variation in water loss rates, variation in the degree of alteration of water availability, and variation in the water limitations of the experimental environment. It is likely that some studies may have failed to find effects of water limitation or stress simply due to a lack of sufficient restriction of water availability or due to water variation being confounded by other factors, for example, by food availability.

Only a small number of studies have examined the effects of variation in water or water stress on animals and those mentioned above are most of the studies with growth as a response. The dearth of studies may be due to difficulties in studying water availability, the belief that water is not limiting in the environment of interest, or the assumption of water's universal importance, causing a lack of interest. Whatever the reason, few studies bother to explicitly consider the effects of water availability on animals, despite its potential importance.

Here we test whether water stress influences growth under laboratory conditions, by altering the hydration states of juvenile domestic house crickets, *Acheta domesticus* L. (Orthoptera: Gryllidae). We hypothesized that dry mass growth rates will increase with increasing hydration state. This study has wide-ranging implications, both for animal care and for field studies of water stress. For instance, if water limitation depresses individual growth rates, it may also limit the size of populations via reductions in survival as shown by Benrey and Denno (1997) and fecundity via increases in time till maturity (Lampert and Trubetskova 1996), which may have other significant impacts, for example, altered resources for predators.

## Study Species

Domesticated *A. domesticus* are generalist consumers and are commonly used in laboratory studies and as feeder species in the pet industry (Walker and Masaki 1989; Clifford and Woodring 1990). Life cycles in this species are relatively short; eggs hatch approximately 13 days after laying if kept moist, larvae take approximately 45 days to reach adulthood and go through 8–9 instars, and adult lifespan is approximately 70 days (Clifford and Woodring 1990). In this study, *A. domesticus* was used as a general model organism for all crickets. With respect to water relations, this species may be representative of many arthropods and a wide range of ectotherms. While it is true that ectotherms have diverse mechanisms of dealing with water stress and tolerances of that stress (Minnich 1982; Hadley 1994), it is likely that when sufficiently stressed, many organisms may experience similar physiological and growth responses (Alpert 2006).

## Materials and Methods

### Hydration state alteration

A pilot study was conducted prior to experimentation to test whether hydration state could be altered by manipulating the number of hours per day with surface water available. For this experiment, juvenile *A. domesticus* were purchased from a local pet store. They were provided with food (Teklad rodent diet 8604, Harlan, www.harlan.com) and water (Fluker's Cricket Quencher Original Formula Water Cubes, www.flukerfarms.com) *ad libitum* and kept under constant temperature (26.7 °C), consistent humidity across treatments (but not necessarily across time), and a 12:12 L:D cycle in an environmental chamber (Conviron CMP 3244, www.conviron.com) for a two day acclimation period. Crickets were then isolated in small plastic containers with ventilation holes and provided with egg carton pieces as shelter and food *ad lib.* Crickets were randomly separated into three levels of water availability: 4, 8, and 24 hours of water available each day (n = 10, 10, and 8 respectively). Crickets were maintained under these conditions for one week. At the end of that week, directly before water replacement would typically occur (at maximum dehydration), crickets were weighed (e = 0.01 g) (Mettler Toledo PG603-S, www.met.com), killed, dried at 60 °C for 48 hours, and reweighed to determine wet mass, dry mass, and hydration. Residuals were roughly normally distributed (as determined by Normal Probability Plots using Systat 10™) and had equal variance (Bartlett's p > 0.995) and thus were analyzed using parametric ANOVA. The treatments significantly altered cricket hydration state (see results).

### Cricket growth

For the growth rate experiment, 111 *A. domesticus* were hatched and reared in shared cages for about 3 weeks. During this period, these crickets received the same conditions as during the acclimation period of the pilot experiment discussed above. At the end of the first 3 weeks, 12 crickets from the reared sample were randomly selected for measurements of initial wet mass, dry mass, body length, and hydration. Because determination of dry mass and hydration requires killing crickets, it was not possible to measure individual cricket dry masses before and after experimental treatments. Therefore, these 12 crickets were assumed to be representative of the population of crickets before experimental treatments were applied. Since all crickets were reared together from eggs and were approximately the same age (+/- 2 days), this assumption is likely reasonable. Further, one would expect that if this is a reasonable assumption, the variation in this initial subsample would be equivalent to the variation within each sample of crickets at the end of experiment. Indeed, the Feltz-Miller test for equality of coefficients of variation (Zar 1999) showed no significant differences between this initial sample and final samples from the three treatment levels (p > 0.1 for each response variable, see results for details; variances were unequal, as might be expected if treatments altered the magnitude of the means).

**Table 1.**
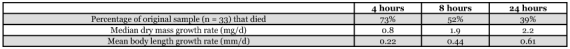
Mortality and average growth rate for crickets in each treatment level.

Remaining live crickets were then individually isolated in small plastic containers with ventilation holes and provided with shelter (egg carton pieces) and food *ad lib*. This prevented scavenging and aided in identification. Due to time constraints, crickets in this experiment were not acclimated to being isolated in cages before beginning treatment application. Crickets were randomly separated into three levels of water availability: 4, 8, and 24 hours of water available each day (initial n = 33). Water was added at 8 am every day and removed after 4, 8, or 24 hours (the 24 hour treatment had its water replaced each day at 8 am). Crickets were reared for 10 days under experimental conditions. None reached adulthood during this experiment. Near the beginning of the experiment there was substantial mortality and only 45 remained at the end of the experiment (4 hr n = 9, 8 hr n = 16, 24 hr n = 20, Table 1). The remaining crickets were measured (length, from tip of head to tip of abdomen) and weighed (wet mass). They were then killed by freezing overnight and dried at 60 °C for 48 hours, allowing measurement of dry mass. Differences in lengths and masses between treatments were assumed to translate into differences in growth, as before and after measurements could not be taken on individuals, since calculation of dry mass requires destructive sampling.

**Table 2.**
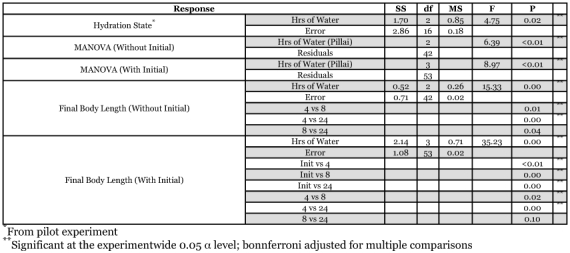
Results of one-way ANOVA of the effects of number of hours per day with available water on hydration state (from pilot experiment) and MANOVA of number of hours of water available on final dry mass and body length combined. Univariate, post-hoc ANOVA of final body length is also presented, with Tukey post-hoc comparisons between individual treatment levels. All post-hoc comparisons are tested using Bonferroni adjusted alpha (0.025). Results are reported without the initial sample of crickets in the analysis (“Without Initial”) and with (“With Initial”).

For all statistical tests of growth, the initial crickets were included as a separate sample and post-hoc comparisons examined differences between these groups (“With Initial” in Table 2). However, results from tests without initial crickets are also included because these are more appropriate for examining solely the differences in growth between treatments (“Without Initial”). Dry mass and length were measured on the same individuals and were not independent measures of growth (Spearman correlation = 0.87). Therefore, MANOVA was performed using the statistical program R, examining the influence of the hydration treatment on dry mass and length combined. Residuals of body length were normal, but those of dry mass were not normal (normal probability plots using Systat 10™) and transformation of dry mass did not improve normality. Natural log transformation of body length improved congruence with the equal variance assumption, and thus all tests were performed on log transformed body length. Pillai's test statistic is robust to violations of normality and equal variance (Zar 1999), thus this test statistic is reported. Post-hoc Kruskal-Wallis one-way ANOVA was performed for dry mass (due to its non-normality and unequal variance, Bartlett's p < 0.001) and parametric ANOVA for log body length. For comparisons of dry mass between treatment levels the post-hoc Dunn test (Zar 1999) was used, and for body length Tukey's method was used. Since dry mass and body length are not independent, for all post-hoc comparisons, the Bonferroni α level was corrected to α = 0.025, making p < 0.025 the criterion for rejection of the null hypotheses.

**Figure 1.**
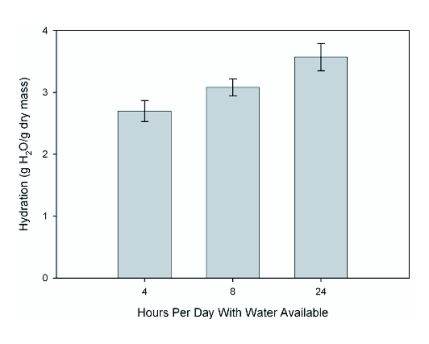
The effects of altered experimental water availability on hydration state. Differences between treatments were significant (F = 4.749, df = 2, 16, p = 0.024).

## Results

### Hydration State Alteration

Differences in the number of hours with free water available resulted in significant differences in cricket hydration state (p = 0.024, F = 4.749, df = 2, 16; Figure 1, Table 2). Crickets with water available for 24 hrs were a mean of 32% more hydrated than crickets with water available for only 4 hrs per day. This is about double the 17% average difference in hydration state observed for field crickets (*Gryllus alogus*) along the San Pedro River in southeastern Arizona (McCluney and Sabo, unpublished data).

### Cricket Growth

**Figure 2.**
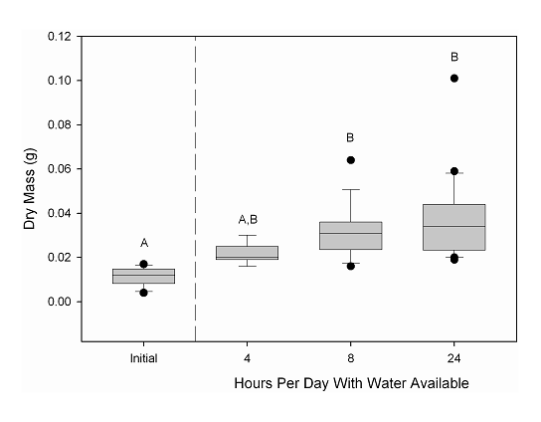
The effects of altered experimental water availability (and thus hydration) on final dry mass. Overall differences between samples were significant (H = 33.846, df = 3, p = 0.000). Letters denote post-hoc significant difference between samples using the Dunn method.

**Figure 3.**
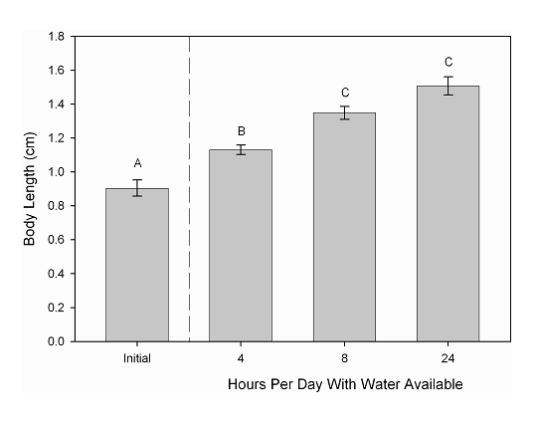
The effects of altered experimental water availability (and thus hydration) on final body length. Overall differences between samples were significant (F = 35.229, df = 3,53, p = 0.000). Letters denote post-hoc significant differences between samples using Tukey's method.

Cricket dry mass and body length combined were significantly influenced by water availability (With Initial: Pillai = 0.67, F = 8.97, df = 3, 53, p < 0.000; Without Initial: Pillai = 0.47, F = 6.39, df = 2, 42, p < 0.000; Table 2; where “With Initial” refers to analysis including the sample of crickets collected at the beginning of the experiment and “Without Initial” is only the three final cricket samples). Post-hoc tests revealed that cricket dry mass was significantly affected by water availability (With Initial: H = 33.846, df = 3, p = 0.000; Without Initial: H = 9.928, df = 2, p = 0.007). There was a significant (Q = 3.16, k = 3, 0.002 < p < 0.005) 59% difference in median dry mass across treatments (Figure 2). Further, median cricket dry mass for crickets with 24 hrs of water was 183% higher at the end of the experiment than initially (Q = 5.45, k =4, p < 0.001), while crickets with water available for only 4 hrs were not significantly higher than at the onset (Q = 2.24, k = 4, 0.2 > p > 0.1). Using median dry mass initially and at the end and dividing by the number of days in the study (10), crickets with 4 hours of water available had dry mass growth rates of 0.8 mg/d, 1.9 mg/d for 8 hrs, and 2.2 mg/d for 24 hrs (Table 1). Post-hoc tests of cricket body length revealed a significant effect of water availability (With Initial: F = 35.229, df = 3, 53, p = 0.000; Without Initial: F = 15.3, df = 2, 42, p = 0.000; Table 2). Post-hoc Tukey's comparisons revealed significant differences between all groups except 8 and 24 hours of available water. There was a 74% difference in mean body length across treatments (Figure 3; p = 0.00). Mean cricket length for crickets with 24 hrs of water was 66% higher at the end of the experiment than initially (p = 0.00), while crickets with water available for only 4 hrs were only 24% higher than at the onset (p < 0.01). This translates into mean body length growth rates of 0.22 mm/d for crickets with 4 hrs of water available, 0.44 mm/d for 8 hrs, and 0.61 mm/d for 24 hrs (Table 1).

The initial subsample of 12 crickets prior to the application of experimental treatments did not have a coefficient of variation that significantly differed from each treatment sample at the end of the experiment, nor did the coefficients of variation between treatment samples differ. There was no significant difference in coefficients of variation for wet mass (0.5 > p > 0.25), dry mass (0.25 > p > 0.1), body length (0.25 > p > 0.1), or hydration (0.5 > p > 0.25).

## Discussion

Results from this study provide strong evidence that water availability and its effects on hydration state can greatly alter individual growth rates. Both dry mass and body length were affected. Further, differences in growth rates in this study, attributable to variation in water availability, were seemingly higher than that from variation in the quality of the diet (Kaufman et al. 1989), although comparisons are difficult given that dry mass growth was not reported (no other studies appropriate for comparison were found). Although limited comparisons could be made, comparisons with Kaufman et al. (1989) do underlie the importance of water for maintaining a physiological state conducive to growth.

While lack of replication prevents a statistical test, the seemingly large differences in survival between hydration state treatments is worth noting (Table 1), and further emphasizes the potential importance of water as a limiting resource for crickets. However, high death rates (39%) of crickets in the 24 hr, high hydration treatment, may indicate that other, non-water related stressors may have been important in our experiment. In fact, the crickets were not acclimated to cages before beginning the experiment, and a large amount of cricket death in all cages occurred in the first 24 hours of the experiment. Further, young house crickets are known to have low survival (Bate 1971). It may have been possible to reduce mortality by increasing the relative humidity. Unfortunately, maintaining a high, constant relative humidity in Arizona is difficult, especially using our environmental chamber, which was not equipped to do this. Therefore, increasing and maintaining constant relative humidity was beyond the equipment available for this study. Increasing the humidity would also have reduced the ability to alter hydration states of crickets.

If the observed patterns of death rates are robust, it provides some supporting evidence for the slow growth-high mortality hypothesis (Clancy and Price 1987; Benrey and Denno 1997). This hypothesis states that slow-growing arthropods often experience higher death rates. However, the mechanism most often proposed is that slower growth increases the time an arthropod is vulnerable to be parasitized or preyed upon. If decreased hydration were a direct source of increased mortality in our study, then this would be an alternative mechanism, where slow growth and high mortality are correlated through physiological water stress.

It was noted that some adult female crickets in the 24 hr, high hydration treatment, had much longer ovipositors (R. Date, personal observation). Since we were unable to determine the sex of the young crickets used in this study at the onset of the experiment, there were very small final sample sizes of females at the end. Therefore, results of statistical tests are not reported here. Future investigation of the effects of hydration on ovipositor length is warranted. Ovipositor length could be an important variable, especially in arid environments, since laying eggs at a lower, moister soil depth may increase reproduction success.

Under field conditions, a variety of other factors, such as favorable microclimates and moist food, may reduce the influence of surface water availability on growth and survival. However, McCluney and Sabo (unpublished data) found large differences in hydration states of crickets at different distances from surface water along the San Pedro River in southeastern Arizona, suggesting the applicability of the laboratory results to field conditions.

There is one particularly important caveat that must be considered for this study. The necessity of killing crickets for measurement of dry mass prevented before/after measurements on individual crickets. Instead a subsample taken at the beginning of the study was compared to the samples at the end of the experiment. Despite the similarity in rearing conditions and coefficients of variation between these samples, it is still possible that there was some bias in the growth of individuals that died versus those that survived. If individuals that grew faster were more likely to die in treatments with lower water availability, it might appear as if these treatments had lower growth rates. However, if this were true it would still result in lower population average growth rates with lower water availability since those that grew faster would be more likely to die, leaving only those that grew more slowly under drier conditions.

Crickets are invertebrate ectotherms and thus these results are only directly applicable to similar organisms. However, the generality of the effects of water stress on growth is illustrated by the range of animals in which water stress has been shown to negatively impact growth (e.g. snapping turtles (*Chelydra serpentina*), McKnight and Gutzke 1993; fat sand rat (*Psammomys obesus*), Kam and Degen 1994).

Variation in growth rates of animals can have far reaching ecological consequences. For example, a faster growth rate may result in earlier maturity and reproduction, and thus faster population growth (Lampert and Trubetskova 1996). Polis (1999) suggests that water limitation may decrease terrestrial animal population growth, and that this may in part explain variation in the prevalence of trophic cascades between aquatic and terrestrial systems. Further, variation in growth rates may alter the distribution of body size, which itself may alter interactions with competitors, predators, and prey (Werner 1994). Finally, variation in growth rate generally influences rates of consumption, and potentially excretion, and may thus alter populations of prey species as well as rates of decomposition and nutrient cycling. For example, more hydrated crickets may consume more dry food as ecological stoichiometry might predict (Sterner and Elser 2002).

There are several environments and conditions where animals are likely to encounter water stress. Organisms living in dry environments, currently 1/3 of the earth's land mass (Schlesinger et al. 1990), may often experience periods of water stress, despite adaptations to reduce this stress, due to the extreme severity of the environment. However, since water stress is simply a desiccating imbalance between water inputs and losses, many relatively wet environments may become extremely challenging during droughts, especially because many of the locally abundant organisms may have few adaptations for maintaining water balance in the face of such challenges. A similar situation might be found along bodies of water that dry seasonally, or are drying with increasing frequency due to climate change or human consumption or alteration such as by constructing dams. In fact, Huxman et al. (2004), show that for plants, all terrestrial areas may be water limiting at some time, for example, during droughts. This may also be true for animals, although perhaps less so, due to their enhanced mobility, allowing for greater water foraging. The variety of negative impacts of water limitation and stress indicate that this resource may be of primary importance to terrestrial animals and thus may often be the most limiting.
